# Treatment of Diabetes

**Published:** 1867-09

**Authors:** Abbots Smith


### DIABETES.

### TREATMENT OF DIABETES.

BY ABBOTS SMITH, M.D., M.R.C.P., &c.

Dr. Smith says, in his instructive little work *On Diabetes*, that the plan of treatment most likely to prove beneficial in this complaint is as follows:—To limit the patient's diet to such articles as do not contain sugar or starch; to attend carefully to the state of the secretions, especially those of the bowels and skin, promoting the action of the former by the administration of podophyllin (so as to act particularly upon the liver), or saline purgatives, and increasing the action of the skin by diaphoretics, the hot-air bath, warm clothing (flannel worn next the skin), and moderate exercise. When the general condition of the patient has in this manner been improved, we must specially direct our attention to the morbid secretion of the urine, and endeavor to check it by suitable medicines. To give any particular remedy, simply because it has been known to be beneficial in diabetes, before the general points referred to have received attention, is opposed to common sense as it is likely to result in failure. The following general diet may be adopted by patients suffering from diabetes:—

*Breakfast.*—Bacon, mutton-chop, or eggs; one of the substitutes for ordinary bread; butter; tea, coffee, or cocoa, made with freshly ground nibs, and not with the ground cocoa-powder, as sold in the shops, unsweetned with sugar, and without milk.

*Dinner.*—Beef-tea, broths, or soups not flavored with carrots or other vegetables; mutton or beef, poultry or game, and fish; cabbages, greens; occasionally, but sparingly, rice pudding without milk, blanc-mange made with cream, not with milk; cheese, butter, and bran, gluten, or almond bread. (This meal should not be taken later than three or four P.M.) For desert, the patient may be allowed a glass or two of sherry or claret, and oily nuts, such as hazel-nuts or filberts, or walnuts. All other kinds of fruit must be interdicted.

*Tea.*—Similar to breakfast, with the exception of meat, which is not requisite.

*Supper* (at nine or ten P.M.).—Dietetic bread and butter, with a little meat, or a small basin of rice milk without sugar.

In the medical treatment of diabetes, the remedies which are of most frequent benefit are alteratives and tonics, so as to improve the appetite and general condition of the patient, and thus enable him the better to bear up against the great drain upon the system, caused by the elimination of the morbid saccharine materials. Quinine, gentian, and other bitters are very useful for improving the tone of the stomach; and the state of the patient may be still further amended by the administration of cod-liver oil, and of the preparations of iron. No remedy in the Materia Medica is of such value in the treatment of diabetes as cod-liver oil, it has a great tendency to improve the condition of the blood by increasing the proportion of the red corpuscles, which undergo considerable diminution in the blood of diabetic persons.

